# Learner-driven innovation in the stone tool technology of early *Homo sapiens*

**DOI:** 10.1017/ehs.2020.40

**Published:** 2020-07-10

**Authors:** Jayne Wilkins

**Affiliations:** Australian Research Centre for Human Evolution, Environmental Futures Research Institute, Griffith University, 170 Kessels Road, Nathan, QLD 4111, Australia; and Human Evolution Research Institute, University of Cape Town, South Africa

**Keywords:** Lithic technology, Middle Stone Age, social learning, innovation, cultural transmission

## Abstract

Current perspectives of stone tool technology tend to emphasize homogeneity in tool forms and core reduction strategies across time and space. This homogeneity is understood to represent shared cultural traditions that are passed down through the generations. This represents a top-down perspective on how and why stone tools are manufactured that largely restricts technological agency to experts, adults and teachers. However, just as bottom-up processes driven by children and youth influence technological innovation today, they are likely to have played a role in the past. This paper considers evidence from the archaeological record of early *Homo sapiens*’ lithic technology in Africa that may attest to our long history of bottom-up social learning processes and learner-driven innovation. This evidence includes the role of emulative social learning in generating assemblages with diverse reduction strategies, a high degree of technological fragmentation across southern Africa during some time periods, and technological convergence through the Pleistocene. Counter to some perspectives on the uniqueness of our species, our ability to learn independently, to ‘break the rules’ and to play, as opposed to conforming to top-down influences, may also account for our technological success.

## Graphical abstract

**Figure:**
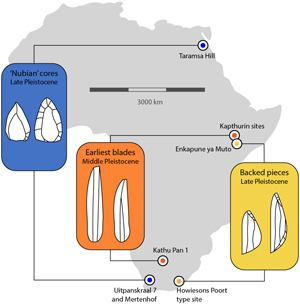

**Media summary:** Lithic technological variability in Pleistocene Africa attests to our long history of non-conformity and bottom-up social learning.

## Introduction

Humans are unusual among extant primates for our heavy reliance on social learning (Tomasello et al., 1993). By at least 3.3 Ma, our hominin ancestors were making and using stone tools to aid in the acquisition of resources (Harmand et al., 2015). Stone tool production creates a large amount of durable debris, and this debris is often the only surviving record of past human behaviours, thus, stone tool technology is the main avenue available to archaeologists for empirically investigating the evolution of human social learning (Stout & Hecht, 2017; Stout, 2018; Shea, 2006; Ranhorn et al., 2020). Some of the earliest stone tools hint that our capacities for social learning via imitation have deep roots in the Plio-Pleistocene (Stout et al., 2019), with more enhanced capacities evidenced in the complex technologies associated with the emergence of our species, *Homo sapiens* (Wadley, 2010; Brown et al., 2009, 2012; Mourre et al., 2010; Wilkins et al., 2012).

One advantage of social learning is that it facilitates cumulative culture change (i.e. the ‘rachet effect’), which is the human capacity to build on the cultural behaviours of others, allowing increases in complexity and/or efficiency over time (Tomasello, 2016; Tennie et al., 2009, 2016; Boyd & Richerson, 2005; Richerson & Boyd, 2005). In consideration of human social learning, much emphasis has been placed on our capacity for high-fidelity information transfer from one generation to the next and the role that active teaching plays in ensuring accurate down-the-line transmission of knowledge (Boyd & Richerson, 2005; Richerson & Boyd, 2005; Tennie et al., 2009; Gärdenfors & Högberg, 2017; Shipton, 2019; Hiscock, 2014; Sterelny, 2012; Henrich, 2017; Shennan, 2002; Tehrani & Riede, 2008). In response, lithic experimental studies have largely focused on skill transmission. For example, it has been shown that teaching through gesture and/or verbal language improves skill transmission, improving flake quality and knapping efficiency in novice knappers (Morgan et al., 2015; Putt et al., 2014, 2017; Cataldo et al., 2018). Interestingly, verbal instruction appears to actually hinder skill development in handaxe reduction; teaching through gesture alone is sufficient for the production of high-quality bifaces (Putt et al., 2014). The first skills that novices need to learn include how to position the core and exploit appropriate core angles (Geribàs et al., 2010), and to control the force of each blow (Bril et al., 2010). With respect to tool form, the opportunity to imitate the manufacturing process appears to reduce ‘copy errors’ and results in more homogeneity in final tool shape (Schillinger et al., 2015, 2016). Pargeter et al. (2019) have developed a skill metric for handaxe production based on multiple measurable variables that include symmetry, flake scar density, and thickness. Knapping activates regions in the brain associated with spatial awareness, strategic action planning, working memory, and language (Stout et al., 2008, 2015; Stout & Hecht, 2017), suggesting that our capacities for knapping may have co-evolved with the other elements of complex cognition and sociality that characterize our species (Stout, 2018). Ethnographic studies focused on lithic technology have emphasized the role of long-term apprenticeship in the production of specific tool forms, while further demonstrating how lithic production can be complexly entwined in the social lives, customs, and epistemologies of the artisans (Weedman, 2006; Stout 2002; Arthur, 2018).

These previous studies have focused almost exclusively on the top-down vertical transmission of knowledge from expert to novice knapper. There has been less consideration of other directions of information flow or how new technologies are introduced and spread, despite innovation being a key component of cumulative culture change (Charbonneau, 2015; Riede et al., 2018). In lithic studies, research context may partially explain this bias. Academic lithicists often learn how to produce stone tools in an academic setting (Shea, 2015; Clarkson, 2017; Eren et al., 2010; Patterson, 1981), where active teaching is standard practice and high-fidelity information transfer is rewarded with formalized evaluation systems. This expert-novice dynamic also characterizes the historical trajectory of lithic studies, in general, and indeed many academic disciplines. It was the highly respected expert knappers François Bordes, Don Crabtree and Jacques Tixier, in the 1950s and 1960s, who set the foundation for many of the technological and experimental approaches that characterize lithic studies today (Kooyman, 2000; Whittaker, 1994; Andrefsky, 2005; Johnson, 1978). Academic lineages linked to these founders is a point of pride for many lithicists. Thus, in the context of a formalized learning institute and WEIRD (Western, Educated, Industrialized, Rich and Democratic) society (Henrich et al., 2010), students of lithic technology and knapping learn early on that the most valuable knowledge is transmitted vertically down the line from teacher to learner.

This perception of how knapping is learned, which might reflect Western, patriarchal values, has influenced not only the experimental studies discussed above, but also how we interpret the archaeological record. First, lithic analysts tend to emphasize homogeneity, looking for similarities across space and time to define industries (Clark et al., 1966; Bishop & Clark, 1967), techno-complexes (Lombard et al., 2012) and ‘named stone tool industries’, or NASTIES (Shea, 2014), and assuming that more similarity represents more interaction, more teaching, more cultural transmission, more top-down transmission of knowledge. Because of this, the concept of standardization became value-laden, and researchers have critiqued the way in which standardization has become problematically linked to cognition, language, and cultural sophistication (Marks et al., 2001; Chazan, 1995; Nowell, 2000). Second, lithic analysts often present one dominant *chaîne opératoire* for an assemblage or type of technology (e.g. Soriano et al., 2007, 2015; Wurz, 2002). Variability is often presented as the unintended consequence of raw material peculiarities, or novices, or other factors that effectively represent ‘noise’ or errors (e.g. Schillinger et al., 2016).

## More than top-down transmission of knowledge

Human social learning is more complex than lithic studies tend to acknowledge. We rely on much more than just down-the-line knowledge transmission via active teaching. Most skill-learning requires both implicit and explicit learning; implicit learning does not rely on conscious efforts and is a foundation process for acquiring complex, tacit knowledge (Seger, 1994; Reber, 1989). Human skill acquisition involves the interaction of two learning pathways: top-down learning (from explicit to implicit knowledge) and bottom-up learning (from implicit to explicit knowledge) (Sun & Zhang, 2004). Of course, cultural transmission can occur both inter- and intra-generationally, vertically, obliquely or horizontally (Boyd & Richerson, 2005; Richerson & Boyd, 2005; Shennan, 2002). Learner skepticism is an important aspect of social learning in humans; learners actively evaluate teachers and will avoid copying those they identify as poor models (Kline, 2015; Boyd & Richerson, 2005). Technological innovation can be used to manifest new identities in opposition and in reference to the status quo (e.g. see discussion of skeuomorphs by Frieman, 2013). Innovation relies just as much on modification generating processes and individuals breaking from the status quo as it does transmission (Charbonneau, 2015). Indeed, the human capacity for rapid cultural change, as opposed to cultural conservation, can offer advantages in variable environments (Potts, 1998). Thus, in some situations, one might consider the most successful learners to be those that do not conform to top-down influences but are active innovative agents that may ‘flip-the-script’ and end up teaching the teachers.

From a traditional perspective of human social learning, learners and novices are children and youth. In accounts of anthropologists and historians, teaching (specifically, student-centred, developmentally appropriate instruction by dedicated adults) plays a minor role in child development outside WEIRD societies (Lancy, 2010). In fact, student-centred teaching between adults and children is so rare that Lancy (2010) suggests that ‘teaching has been largely superfluous in the process of cultural transmission through human history’ (p. 97), except for the very recent past. Rather, children learn by being immersed in the activities of their society, and with various members of that society assisting through opportunity provisioning, evaluation and increasing accessibility (an ethological definition of teaching, Lew-Levy et al. 2020). For example, hunter–gatherer children learn several kinds of skills, including subsistence-related skills, from other children, not just the adults in their social network (Lew-Levy et al., 2017, 2020). As part of the developmental process, children copy some adult behaviours, often through play and sometimes with miniatures – small imitations of material culture, examples of which have been observed ethnographically and archaeologically (Langley & Litster, 2018). Through play, children and youth explore and test the boundaries of materials, systems, technologies, and language, and in this way, play not only serves a role in learning, but can also can act as a primer for innovation (Riede et al., 2018; Nowell, 2016; Pellegrini et al., 2007; Langley et al., 2020). In other words, children and youth do not simply absorb and recreate the behaviours of the adults around them. They learn independently, they learn from other children, and thus, they can also be active, innovative agents of technological and societal change.

**Figure 1.**
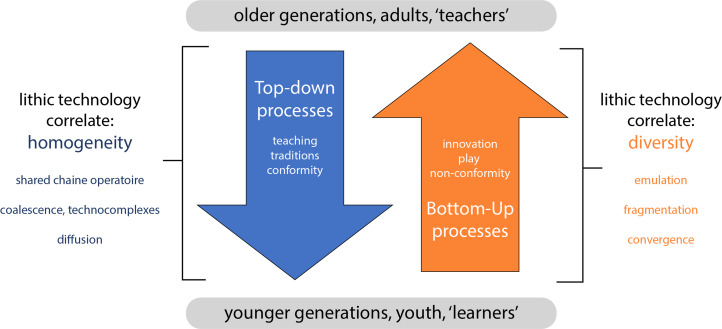
Summary of bottom-up vs. top-down social learning processes and lithic technological correlates proposed here.

The purpose of this paper is to explore learner-driven innovation in lithic technology. Innovation requires a new idea that is subsequently transmitted. The general perception in lithic studies is that the source of the new idea is the expert, adult or teacher, but the research summarized above shows that the source can also be the novice, child or learner. Learner-driven innovation is facilitated by what I describe as bottom-up social learning processes – actions that result in knowledge transfer up the chain, from, for example, novices to experts (Figure 1). This contrasts with top-down social learning processes that result in knowledge transfer down the chain, from experts to novices, which is the emphasis of most previous considerations of social learning and lithic technology. Here, I consider how the archaeological record can inform us of past human social learning environments, which may help direct future experimental research. I suggest three ways learner-driven innovation may be expressed archaeologically and consider examples of this for the African Middle Stone Age (MSA, ~500–40 ka), which is critical for understanding the behavioural evolution of early *Homo sapiens*. The three archaeological correlates of learner-driven innovation that I suggest here are: *emulation* – applying self-determined strategies to accomplish a shared end goal; *fragmentation* – high diversity across geographic space; and *convergence* – independently invented similarity (Figure 1). These correlates are not isolated illustrations of individual innovation events, but rather the results of processes that over the long term led to the archaeological patterns of the African MSA that attest to our human capacity for learner-driven innovation.

## Emulation

Although sometimes used colloquially as synonyms, emulation and imitation differ with respect to process. Imitation occurs when learners copy the exact procedures and strategies of others for accomplishing a shared end goal (process copying), while emulation occurs when learners employ different strategies for accomplishing that shared end goal (product copying, Schillinger et al., 2015; Caldwell & Millen, 2009; Tennie et al., 2009; Bentley, 2018; Whiten et al., 2009). Both imitative and emulative forms of learning are part of the ‘portfolio of different social learning processes’ employed by humans, chimpanzees (Whiten et al., 2009), and by extension, probably all species within the hominin lineage. Under some conditions, human children, but not chimpanzees, overimitate – that is, they copy actions that are casually irrelevant with high fidelity (for a review see Whiten et al., 2009). In part owing to this difference between chimpanzee and human children, considerations of Palaeolithic and Stone Age technologies have almost exclusively focused on imitation, and the human capacity for imitation has been used to explain cumulative culture change (Boyd & Richerson 2005). However, whether human children overimitate depends on several factors, which include whether the model is constrained to act that way or not (Gergely et al., 2002), and whether the actions can be interpreted as accidents (Carpenter et al., 1998). Emulation can also lead to cumulative culture change in the absence of imitation (Caldwell & Millen, 2009; Wasielewski, 2014). Yet the role of emulation in the past as reflected in lithic technology, and its role in understanding cumulative culture change in the Pleistocene, is rarely examined.

There are several varieties of emulation (Subiaul et al., 2016; Whiten et al., 2009). One main distinction directly relevant to this discussion is whether the actions and techniques are observable or not. In *reverse engineering*, or end-state emulation in the absence of a demonstration, only the finished product is observed, and there is no opportunity to observe how the actions are carried out. In *goal emulation*, the actions of another carrying out or attempting to carry out the goal are observed.

Both types of emulation are considered a reflection of learner-driven innovation here, because the learner develops their own strategies for accomplishing a goal, rather than relying on top-down transmission of those strategies. Emulation still involves copying a model – ideas about what to produce are shared – but exactly how those goals are accomplished can vary significantly between the novice and the expert. Through emulation, novices generate new technological procedures that others within the social network may subsequently uptake via imitation. Emulation as a social learning strategy may be advantageous in certain situations (Wilkins, 2018). First, emulation relies on little to no teaching effort from experts, which frees up time for experts to pursue other goals. Second, because the novice is employing trial-and-error learning on the procedures used to accomplish a goal, it may lead more quickly to effective solutions on new or variable raw materials that the expert has not regularly encountered. Lastly, it generates diversity on which selective processes can act, leading to the transmission of innovations and inventions.

Based on experimental studies, lithic assemblages resulting from reverse engineering have end products that exhibit higher degrees of shape variability (Schillinger et al., 2015; Ranhorn, 2017) and core reduction strategies (Ranhorn, 2017). Lithic reduction experiments focused specifically on goal emulation have not yet been reported. However, in an experiment where novices learned how to make handaxes in verbal and non-verbal settings, those in the non-verbal setting tended to emulate the demonstrator, rather than imitate (Putt et al., 2014). While there were few differences between the resulting handaxes from the two conditions, the non-verbal condition where novices knappers tended to emulate resulted in debitage reflecting higher levels of flaking efficiency (i.e. large, thin flakes), but also more debitage. Participants in the verbal condition tended to imitate the production of ambitious platforms that they did not yet have the skill to execute, and this resulted in debitage reflecting lower levels of flaking efficiency (i.e. large, thick flakes), but also less debitage (Putt et al. 2014).

Reverse engineering requires no direct interaction between individuals, while goal emulation, in contrast, occurs in social settings. Based on what we know about early human sociality (Gamble, 1999), there would have been many opportunities for goal emulation through the Pleistocene, and indeed for the whole portfolio of social learning processes available to early humans. For example, over the course of their lifetime, individuals witness others making large, regular points for their spears, and they may witness experienced knappers using bifacial, hierarchical cores to do so. Through a combination of observation and trial-and-error learning across many unstructured learning sessions, they develop their own strategy for making large, regular points, which is similar to, but also different from the model knappers around them. In this sort of context, it is likely that some active teaching and imitation also occur at some stages of the learning process, which is why a continuum is appropriate (Bentley, 2018). On the emulation side of this continuum, one expects more diversity in core reduction strategies, and on the imitation and active teaching side of this continuum, one expects less diversity in core reduction strategies. Based solely on the metrics of discarded flakes, one cannot accurately deduce core reduction strategy (Eren et al., 2018); however, experimental research has shown that through a combination of core and flake attributes, it is possible to deduce MSA core reduction strategies (Scerri et al., 2016).

There is empirical evidence from the MSA record of Africa for the application of emulative learning strategies. In the Middle Pleistocene assemblage from Kathu Pan 1, three lines of evidence were used to argue that there was more emphasis on emulation than imitation to produce points discarded there (Wilkins, 2018). First, diverse reduction strategies, including preferential and recurrent Levallois strategies, as well as non-Levallois strategies, were used to produce blades and flakes. Second, both blades and flakes were used to produce unifacially retouched points. Third, diverse point types (unretouched blades and flakes, unifacially retouched blades and flakes) show evidence of having been used as weapon tips. From a top-down perspective, one would expect similarity in the reduction strategies and selective processes employed for manufacturing stone tools, but rather, intra-assemblage diversity suggests that a variety of processes were used to accomplish a single goal – a stone point suitable for use as a weapon tip. While evidence for emulation is rarely explicitly reported, several descriptions of MSA assemblages highlight core reduction strategy diversity (Table 1). This includes the MSA levels at Porc-Epic Cave, Ethiopia (Pleurdeau, 2006), Koimilot, Kenya (Tryon, 2006; Tryon & McBrearty, 2006; Tryon et al., 2005) and Kudu Koppie, South Africa (Wilkins et al., 2010). This intra-assemblage diversity hints that top-down, high-fidelity cultural transmission was not always a priority for MSA knappers.

**Table 1.** Summary of MSA assemblages that document coeval diverse core reduction strategies

Site	Location	Unit and age estimate	End product(s)	Strategies	References
Kathu Pan 1	Southern Kalahari Basin, South Africa	Stratum 4a, ~500 ka (Fauresmith or early MSA)	Retouched points, on blade and flake blanks	Preferential and recurrent Levallois strategies, as well as non-Levallois strategies	Wilkins, 2018; Wilkins & Chazan, 2012
Porc-Epic Cave	Dire Dawa, Ethiopia	Units I–IV, age uncertain (MSA)	Flakes and blades	Four different schemes that include Levallois and non-Levallois strategies, there is no temporal patterning in these schemes through the sequence	Pleurdeau, 2006
Koimilot	Kapthurin Formation, Kenya	Locus 1, ~200 ka (early MSA)	Flakes	Preferential and recurrent Levallois strategies, as well as non-Levallois strategies (‘platform’)	Tryon, 2006; Tryon & McBrearty, 2006; Tryon et al., 2005
		Locus 2, ~200 ka (early MSA)	Points and blades	Blades from platform cores, points from unidirectional convergent Levallois strategy	
Kudu Koppie	Limpopo River valley, South Africa	MKKU, no age estimate (MSA)	Flakes	Diverse Levallois strategies, including preferential, recurrent centripetal, recurrent unidirectional and recurrent bidirectional	Wilkins et al., 2010

## Fragmentation

Various proxies are used by archaeologists to make interpretations about inter-group connectedness in the MSA. MSA stone points and proposed armature tips (i.e. backed pieces as arrowheads) show variability on continent-wide geographic and temporal scales, and it has been suggested that this variability reflects cultural patterns (Clark, 1992; McBrearty & Brooks, 2000; Foley & Lahr, 1997, 2003; Willoughby, 2007; Wadley, 2001). This suggestion is largely based on the potential role that armatures (and other technologies) can play in communicating messages about ethnic or group affiliation (Wiessner, 1983). Armatures and other technologies can also serve as important symbolic objects in regional exchange networks, and thus may reflect cultural inter-connectedness (Wilkins, 2010). The transport distances of stone raw material across the landscape can also shed light on the potential reach of exchange networks for goods and information (Ambrose & Lorenz, 1990). Because knapping is learned in a social environment, reduction strategies and other aspects of lithic technology (beyond point form) can also serve as proxies for inter-group connectedness (Gamble, 1999).

In their meta-analysis of MSA technological variability, Mackay et al. (2014) considered the spatial and temporal patterns of various lithic technological systems (provisioning, raw material selection, flaking systems and implement types) to identify past periods of inter-regional coalescence (interaction) and fragmentation. They suggest that during MIS 4, which is a glacial period dated to ~57–71 ka, there was increased interconnectedness, or ‘coalescence’ of human groups across southern Africa (Table 2). In contrast, during MIS 5 and 3 (interglacials dated to ~71–130 ka and ~29–57 ka, respectively), human populations were more fragmented. In other words, during some periods in the MSA, early human populations did not share lithic technological information across large areas or for long periods of time. Instead, local approaches thrived.

**Table 2.** Summary of population connectedness during the Middle and Later Stone Age in South Africa based on lithic technological evidence, after Mackay et al. (2014)

MIS	Age range	Period	Population connectedness	Summary of evidence
2	14–29 ka	LSA	Coalescence	Bladelet and bipolar technologies show similarity at inter-regional scale (i.e. Wilton and Robberg technocomplexes), non-local raw material movements at regional scale
3	29–57 ka	MSA (LSA toward end of MIS 3)	Fragmentation	Diverse flaking strategies that show regional variability, implement forms (e.g. hollow-based points) have limited distributions, focus on local materials
4	57–71 ka	MSA	Coalescence	Blades and backed artefact technologies show similarity at regional and inter-regional scales (i.e. Howiesons Poort technocomplex), non-local raw material movements at regional scale
5	130–71 ka	MSA	Fragmentation	Focus on local materials

The archaeological record of MIS 3, in particular, shows a mosaic, patchy pattern of technological variability consistent with fragmented populations. In South Africa, assemblages dated to this time variability exhibit evidence for bipolar percussion, bladelets and microliths, formal tool types with repeated forms such as hollow-based points, unifacial points, and scrapers and knives, as well as point and blade-based assemblages with rare retouched forms (Lombard et al., 2012; Wurz, 2013, 2019). Some regional patterns are hinted at, but are difficult to define, and overall this time period shows highly variable lithic technologies across South Africa. A similar pattern of irregular temporo-spatial distribution of lithic assemblage traits also characterizes the Late Pleistocene in East Africa (Ranhorn & Tryon, 2018; Tryon & Faith, 2013).

Fragmentation can be considered a result of learner-driven innovation on a large scale. It reflects weaker homogenizing processes over time and across space, with less social emphasis on imitation, high-fidelity information transfer and the passing down of technological traditions. This is not to say that top-down processes were absent; they must have been present to maintain some intra-assemblage and intra-regional similarities, but the social rules governing top-down information transfer must have also been relaxed enough to permit the innovation and spread of new ideas about lithic reduction.

## Convergence

Humans often converge on similar solutions to similar problems, without cultural transmission. Convergence in stone tool form and reduction strategies has been documented across the world for many time periods (e.g. Kuhn & Zwyns, 2018; Clarkson et al., 2018; Will et al., 2015; Sharon, 2019; Eren et al., 2013, 2018; Wilkins, 2018; Wilkins & Chazan, 2012; Lycett, 2009; Jennings & Smallwood, 2018; Adler et al., 2014), and convergent evolutionary processes in lithic technology are receiving increasingly more attention from methodological and theoretical perspectives (O'Brien et al., 2018; Groucutt, 2020).

Convergence can reflect learner-driven innovation; similar technological solutions result from independent, trial-and-error learning to solve similar problems, rather than through cultural transmission. It can represent a much larger scale than the concept of emulation described above. It can involve individuals, but also groups at various scales. Individuals can converge on a solution, for example, and so can groups or populations.

Archaeologically, convergence can be challenging to empirically demonstrate. Early twentieth-century Diffusionist approaches tended to overinterpret similarity across geographic space as an indication of spreading populations and ideas. Convergence is now more commonly accepted as a potential explanation for similarity in form between very distant geographic locales. Some researchers draw from evolutionary ecology, employing cladistic approaches to examine evolutionary relationships in form and technological characteristics between different assemblages of stone tools (Lycett, 2009; O'Brien et al., 2001; Buchanan & Collard, 2007; O'Brien & Lyman, 2003). This can suggest different ‘lineages’ within populations of stone tools that are superficially similar in form (i.e. projectile points). Technological similarity between assemblages is sometimes used to support arguments for cultural connection. For example, a connection has been made between Clovis and Solutrean assemblages in North America and Europe, respectively. The argument is based on the observation that their characteristic bifacial points were produced using similar reduction strategies (Stanford & Bradley, 2012; Lohse et al., 2014). However, one of the components of this strategy, the removal of overshot thinning flakes, has been argued to be unintentional and occurring in different frequencies between the two assemblages, problematizing the proposed Clovis–Solutrean connection (Eren et al., 2013, 2014).

**Figure 2.**
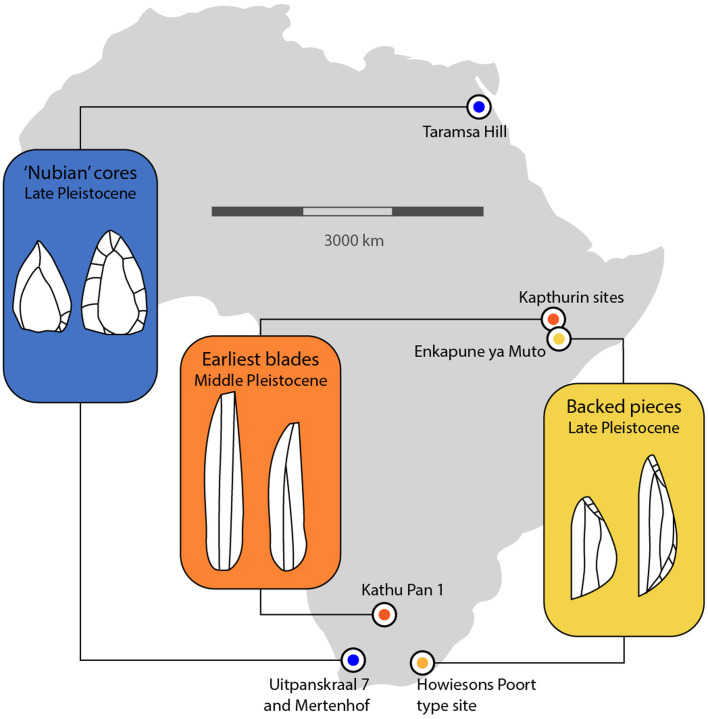
Examples of technological convergence in the African Middle Stone Age.

There are several potential examples of convergence in African MSA technologies (Figure 2). For example, the earliest evidence for blade production occurs roughly coeval in southern (Kathu Pan 1) and East Africa (Kapthurin sites) ~500 ka, and these blades were produced using different reduction strategies consistent with convergence (Wilkins & Chazan, 2012). ‘Nubian-like’ cores for the production of points from the Cedarberg region of southern Africa exhibit morphologies and technological characteristics similar to cores described in Egypt, such as those at Taramsa Hill (Will et al., 2015; Vermeersch et al., 1998). In fact, similar kinds of cores, described generally as Levallois point cores, have been reported at other southern African MSA sites (Thompson et al., 2010; Wilkins & Chazan, 2012; Wilkins et al., 2017; Wurz, 2002). While impossible to prove conclusively, convergence in core reduction strategy is a more likely explanation for similarity between regions ~6500 km apart than cultural transmission. Archer et al. (2016) used three-dimensional geometric morphometrics to examine bifacial point variability in Still Bay-designated assemblages, finding a high degree of variability between points in the north-east and south-west of South Africa. They suggest that convergence is the most likely explanation for the occurrence of bifacial points across the region. ‘Miniaturization’ of stone tools can be advantageous in a variety of circumstances related to raw material availability and mobility, among others (Pargeter & Shea, 2019). The relative importance of the production of bladelets and backed pieces fluctuates through the Late Pleistocene and Holocene across many regions of Africa and beyond (Pargeter & Shea, 2019; Clarkson et al., 2018). Backed pieces are known at Howiesons Poort-designated sites in South Africa dated to ~100–50 ka (Lombard et al., 2012; Porraz et al., 2013; Jacobs et al., 2008; Lukich et al., 2019) and also from Enkapune ya Muto and other sites in East Africa by ~50 ka (Ranhorn & Tryon, 2018; Ambrose, 2002).

## Variability, non-conformity and *Homo sapiens*

The observations presented here highlight lithic technological variation at multiple scales – intra- and inter-assemblage and intra- and inter-regional – in the African MSA. Indeed, variation is the norm in the lithic technology of *Homo sapiens* (Shea, 2011), and its meaning needs consideration as much as homogeneity does. The current literature on cultural transmission and innovation in lithic technology focuses largely on homogeneity, perhaps owing in part to academic biases about knowledge transmission, and is thus inadequate for understanding this variability. I suggest here that learner-driven innovation and bottom-up social learning processes contributed to the observed patterns of variability, and that the archaeological record of early *Homo sapiens* shows abundant evidence for this. Indeed, the Middle Range theory for how technological variations are developed and spread requires more rigorous development, especially through knapping and social transmission experiments (Ranhorn et al., 2020). Based on the variable archaeological record of early *Homo sapiens* discussed here, continued investigations should not continue to focus solely on top-down approaches like active teaching, imitation and high-fidelity cultural transmission. They should also consider why, when and how knappers choose to not conform.

Human behaviour is complex, and it is reasonable to assume that we relied on a variety of learning mechanisms through the entirety of our evolutionary history. There may have been variable emphasis on top-down and bottom-up processes through time and across space, and dependent on the type of technology considered (Wilkins, 2020). For example, the production of backed bladelets and their probable incorporation into high-velocity projectile systems during the Howiesons Poort (Lombard & Pargeter, 2008; Pargeter, 2007; Schoville et al., 2017) may have relied more heavily on high-fidelity information transfer than other kinds of Stone Age technology. Humans knap and produce stone tools in a huge variety of contexts around the world. Top-down social learning processes, at a cost to the teacher, can ensure the transmission of known fitness-enhancing information and skills. On the other hand, independent learning and learner-driven innovation offer some advantages that include reduced teaching costs and quicker rates of change in fluctuating environments. Thus, several factors – the nature of the goal, environmental variability, group and population size, subsistence, settlement, socio-cultural factors – may influence the relative advantages of top-down and bottom-up processes of cultural transmission.

Experimental work has hinted that the relative effectiveness of imitation vs. emulation in lithic reduction is goal-dependent. The results and observations of the skill transmission experiment by Putt et al. (2014) suggest that emulation is as effective as imitation for producing handaxes of a particular form, but that emulation can be *more* effective than imitation for producing large, thin flakes.

Ethnographic research on hunter–gatherer children adds support to the idea that role of adults in teaching is context dependent (Lew-Levy et al., 2020). Child-to-child teaching is more frequent than adult-to-child teaching for many activities in many hunter–gatherer societies (Lew-Levy et al., 2017, 2020). Lew-Levy et al. (2020) found significant differences in teaching between two groups – the Hadza (Tanzania) and BaYaka (Congo). For the BaYaka, 5-year-olds receive almost as many hourly teaching events from other 5-year-olds as they do from 25-year-olds. The same pattern is not true for the Hadza, and the authors attribute this to differences in settlement and subsistence (Lew-Levy et al., 2020).

Learners, children and youth, can be active innovative agents, contributing more than simply acquiring and passing on the knowledge of the generation before them. This must be considered when interpreting lithic assemblage variability and investigating social transmission in the past. From personal experience living in the modern world, we know that some of our richest learning experiences include independent learning, experiential learning, experimentation and creativity. We know that youth often drive technological change, fashion change and social change (e.g. smartphone apps), and we know that younger generations challenge older ones. Counter to the narrative that high-fidelity information transfer defines us, our ability to learn independently, to avoid conformity, and to play, rather than to simply follow top-down influences, also significantly influenced the success of human societies in the past.

## Data Availability

As a conceptual study, this manuscript does not rely on shareable data, code or other resources.
